# Enhanced Production of Botrallin and TMC-264 with *in Situ* Macroporous Resin Adsorption in Mycelial Liquid Culture of the Endophytic Fungus *Hyalodendriella* sp. Ponipodef12

**DOI:** 10.3390/molecules190914221

**Published:** 2014-09-10

**Authors:** Haiyu Luo, Hongwei Liu, Yuheng Cao, Dan Xu, Ziling Mao, Yan Mou, Jiajia Meng, Daowan Lai, Yang Liu, Ligang Zhou

**Affiliations:** 1 MOA Key Laboratory of Plant Pathology, Department of Plant Pathology, College of Agronomy and Biotechnology, China Agricultural University, Beijing 100193, China; E-Mails: luohaiyu69@163.com (H.L.); liuhongweicau@163.com (H.L.); caoyuheng1990@163.com (Y.C.); cauxudan@163.com (D.X.); maoziling2011@163.com (Z.M.); muyan01987@163.com (Y.M.); mengjiajiax@163.com (J.M.); dwlai@cau.edu.cn (D.L.); 2 Institute of Agro-products Processing Science and Technology, Chinese Academy of Agricultural Sciences, Beijing 100193, China; E-Mail: liuyang01@caas.cn

**Keywords:** dibenzo-α-pyrone, botrallin, TMC-264, endophytic fungus, *Hyalodendriella* sp. Ponipodef12, fermentation, macroporous resin, *in situ* resin adsorption

## Abstract

*Hyalodendriella* sp. Ponipodef12, an endophytic fungus from the hybrid “Neva” of *Populus deltoides* × *P. nigra*, is a high producer of the bioactive dibenzo-α-pyrones botrallin and TMC-264. However, both the botrallin and TMC-264 produced by *Hyalodendriella* sp. Ponipodef12 were retained as both intracellular and extracellular products. The aim of this study was to evaluate an *in situ* macroporous resin adsorption for enhancement of botrallin and TMC-264 production in mycelial liquid culture of *Hyalodendriella* sp. Ponipodef12. Production of botrallin and TMC-264 was most effectively enhanced by macroporous resin DM-301 among the thirteen nonionic macroporous resins tested. The highest botrallin yield (51.47 mg/L, which was 2.29-fold higher than the control at 22.49 mg/L) was obtained by adding resin DM-301 at 4.38% (g/mL) to the culture broth on day 24 and allowing a period of 4 days for adsorption. The highest TMC-264 yield reached 47.74 mg/L, which was 11.76-fold higher than that of the control (4.06 mg/L), and was achieved by adding DM-301 resin at 4.38% (w/v) in the culture broth on day 24 and allowing a period of 6 days for adsorption. The results show that *in situ* resin adsorption is an effective strategy for enhancing production of botrallin and TMC-264, and also for facilitating their recovery from mycelial liquid culture of *Hyalodendriella* sp. Ponipodef12.

## 1. Introduction

Plant endophytic fungi are an important and novel resource of natural bioactive compounds with great potential applications in agriculture, medicine and food industry [1,2,3,4]. Many valuable bioactive compounds with antimicrobial, insecticidal, cytotoxic and anticancer activities have been successfully discovered in endophytic fungi during the past few years [5,6,7,8].

Botrallin and TMC-264 are members of the dibenzo-α-pyrone class of compounds (also named dibenzo-α-pyranones, 6*H*-benzo[*c*]chromen-6-ones, and 6*H*-dibenzo[*b*,*d*]pyran-6-ones) [9]. Botrallin has been isolated from the fungi *Botrytis allii* [10], *Microsphaeropsis olivacea* [11] and *Hyalodendriella* sp. [12], and has antimicrobial, antinematodal and acetylcholinesterase inhibitory activities [11,12]. TMC-264 has been isolated from the fungi *Phoma* sp. [13] and *Hyalodendriella* sp. [14]. It also shows antimicrobial and antinematodal activities [14]. In addition, TMC-264 selectively inhibited interleukin-4 (IL-4) signaling by interfering with phosphorylation of the signal transducer and activator of transcription 6 (STAT6), as well as binding of the phosphorylated STAT6 to the recognition sequence, so it might be an inhibitor of IL-4 signaling for treatment of allergic diseases [15].

Both botrallin and TMC-264 have been identified as the main bioactive dibenzo-α-pyrones from the ethyl acetate extract of the endophytic fungus *Hyalodendriella* sp. Ponipodef12 isolated from the hybrid “Neva” of *Populus deltoides* Marsh × *P. nigra* L [12,14]. Furthermore, these two compounds were found both intracellularly and extracellularly in liquid culture of *Hyalodendriella* sp. Ponipodef12. In order to speed up development of their applications, the most important approach is to increase the yield of botrallin and TMC-264 in fermentation culture. Many strategies (*i.e.*, medium optimization, addition of metal ions, elicitation by using polysaccharide and oligosaccharide, as well as two-phase culture) to enhance the production of bioactive compounds in either microorganism or plant cultures have been well developed so far [16,17,18,19,20,21,22].

In order to improve botrallin and TMC-264 production in the *Hyalodendriella* sp. Ponipodef12 fermentation process, a strategy by making use of *in situ* macroporous resin adsorption (or called solid-phase *in situ* product adsorption) in mycelial liquid culture was introduced in this study. The *in situ* product removal by using macroporous resins in fermentation systems is an integrated bioprocess of production and separation. It has many benefits for the overall fermentation process by facilitating the product recovery, eliminating feedback inhibition, overcoming product degradation, avoiding product autotoxicity, reducing cost, and improving the product yield efficiently [23,24]. The strategy by employing *in situ* macroporous resin adsorption has been successfully applied for increasing metabolite yields in many culture systems, such as the hairy root culture of *Salvia miltiorrhiza* for tanshinone production [25], fungal culture of *Fusarium redolens* Dzf2 for beauvericin production [26], fungal culture of *Berkleasmium* sp. Dzf12 for diepoxin ζ production [27], and bacterial culture of *Streptomyces xinghaiensis* for xinghaiamine A production [28].

To the best of our knowledge, there were no previous reports on *in situ* product removal for enhancing production of botrallin and TMC-264 by using solid-phase adsorption in fungal liquid culture. The purpose of this study was to investigate the *in situ* macroporous resin adsorption of botrallin and TMC-264 including the types, concentration, addition time and incubation period of resin for improving the metabolite production in mycelial liquid culture of *Hyalodendriella* sp. Ponipodef12 to meet future development and application needs.

## 2. Results and Discussion

### 2.1. Kinetics of Hyalodendriella sp. Ponipodef12 in Normal Batch Culture

**Figure 1:**
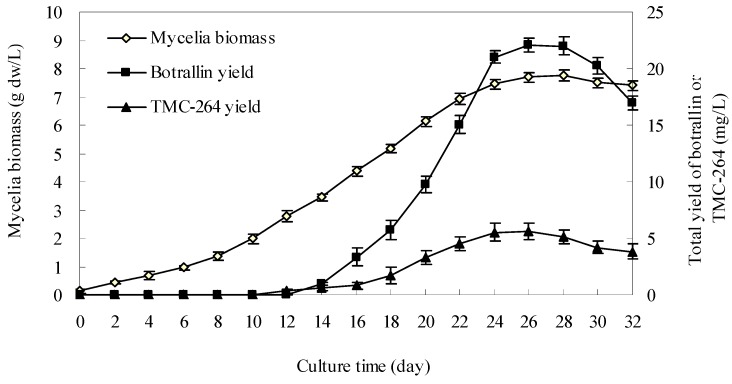
Time courses of mycelial growth, and production of botrallin and TMC-264 in liquid culture of *Hyalodendriella* sp. Ponipodef12 (error bars for standard deviations, *n* = 3).

The time courses of mycelial growth as well as botrallin and TMC-264 accumulation in *Hyalodendriella* sp. Ponipodef12 mycelial liquid culture are shown in Figure 1. The mycelia biomass increased slowly during the first 6 days, then increased more rapidly between day 7 and day 28, and reached the maximum (7.76 g dw/L) around day 28 of culture. The botrallin and TMC-264 accumulation remained at a very low level from day 0 to day 10, and then increased steadily from day 10 to day 26. The maximum yields (intracellular plus extracellular) of botrallin and TMC-264 obtained on day 26 were 22.02 mg/L and 5.63 mg/L, respectively. The results indicated that day 26 was a suitable time to harvest fermentation cultures of *Hyalodendriella* sp. Ponipodef12 for botrallin and TMC-264 production, and also suggested that the resins should be added in medium before day 26 of culture to efficiently adsorb botrallin and TMC-264.

### 2.2. Effects of the Resins on Mycelial Growth and Production of Botrallin and TMC-264

The adsorbents considered for *in situ* adsorption of the target metabolites in fermentation may vary in chemical and physical properties such as polymer chemistry, surface area, particle size and pore size [24]. According to our preliminary results (data not shown), 5.00% (g/mL) should be an appropriate concentration for each resin. The effects of thirteen selected representative macroporous resins at 5.00% (g/mL) on mycelial growth and production of botrallin and TMC-264 in liquid culture of *Hyalodendriella* sp. Ponipodef12 are listed in Table 1. Resins X-5 and DA-201 had no significant effects on the mycelial growth, while other resins (D101, HPD-100, D1300, D3520, AB-8, DM130, DS-8, DM-301, ADS-17, S-8, and NKA-9) exhibited slightly inhibitory effects on the mycelial growth.

**Table 1:** Effects of the resins on mycelial growth, production of botrallin and TMC-264 in liquid culture of *Hyalodendriella* sp. Ponipodef12.

Resin	Mycelia Biomass (g dw/L)	Botrallin Yield (mg/L)	TMC-264 Yield (mg/L)	Yield of Botrallin Plus TMC-264 (mg/L)
CK	7.34 ± 0.03 a	19.52 ± 1.25 cd	3.77 ± 0.43 g	23.29 ± 0.82 def
X-5	7.15 ± 0.22 a	18.61 ± 0.90 d	5.56 ± 0.17 e	24.17 ± 0.82 de
D-101	6.73 ± 0.15 c	16.00 ± 1.83 e	5.85 ± 0.61 e	21.85 ± 1.98 f
HPD-100	6.35 ± 0.29 e	20.14 ± 1.31 c	4.57 ± 0.84 f	24.71 ± 2.10 d
D1300	6.91 ± 0.25 b	21.04 ± 2.08 c	1.98 ± 0.13 h	23.02 ± 2.19 def
D3520	6.94 ± 0.45 b	25.61 ± 0.69 b	4.44 ± 0.27 f	30.05 ± 0.95 b
AB-8	6.60 ± 0.40 c	20.63 ± 0.13 c	4.45 ± 0.02 f	25.08 ± 0.13 d
DM130	6.68 ± 0.29 c	20.15 ± 1.16 c	7.25 ± 0.23 c	27.40 ± 0.92 c
DS-8	6.80 ± 0.46 b,c	14.45 ± 0.20 f	5.35 ± 0.05 e	19.79 ± 0.14 g
DM-301	7.07 ± 0.07 b	27.98 ± 0.01 a	33.86 ± 0.02 a	61.84 ± 0.02 a
ADS-17	6.94 ± 0.16 b	20.30 ± 0.89 c	11.40 ± 0.46 b	31.70 ± 0.49 b
S-8	6.64 ± 0.13 cd	4.62 ± 0.22 g	5.40 ± 0.15 e	10.01 ± 0.30 h
NKA-9	6.48 ± 0.37 de	24.04 ± 0.67 b	6.31 ± 0.34 d	30.35 ± 0.91 b
DA-201	7.26 ± 0.17 a	18.75 ± 0.03 d	3.82 ± 0.43 g	22.57 ± 0.40 f

*Notes*: CK, without addition of the test resin; the concentration of each resin in medium was 5.00% (g/mL); the values are expressed as means ± standard deviations (*n* = 3). Different letters (*i.e*., a, b, c, …) indicate significant differences among the treatments of resins in each column at *p* = 0.05 level.

Botrallin production was obviously enhanced by the resins DM-301, D3520 and NKA-9, and was inhibited by the resins D-101, DS-8 and S-8. Other resins including X-5, HPD-100, D1300, AB-8, DM130, ADS-17, and DA-201 had no significant effects on botrallin production. For TMC-264 production, both DM-301 and ADS-17 resin showed the most enhancing effects with TMC-264 yields of 33.86 mg/L and 11.40 mg/mL, respectively. Only D1300 resin exhibited inhibitory effects.

**Figure 2:**
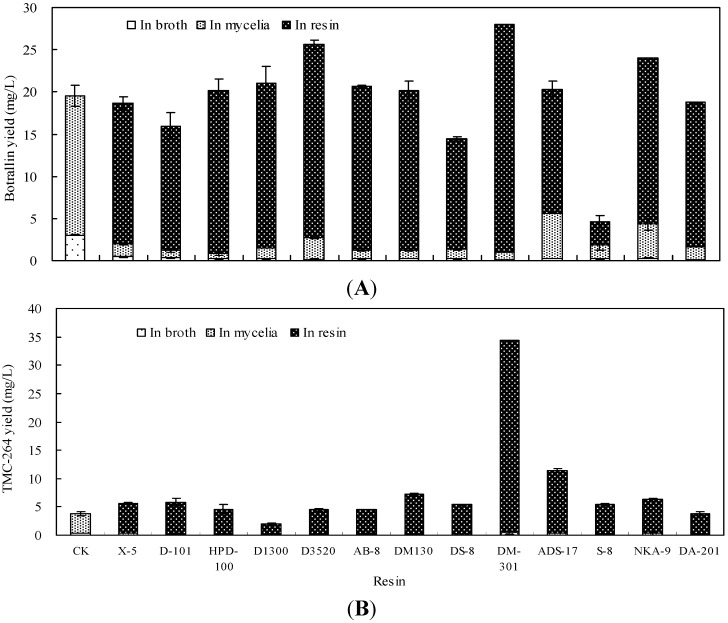
Effects of the resins (5.00%, g/mL) on production of botrallin (**A**) and TMC-264 (**B**) along with their distribution in the broth, mycelia and resins in liquid culture of *Hyalodendriella* sp. Ponipodef12 (error bars for standard deviations, *n* = 3).

The distribution of botrallin and TMC-264 in the broth, mycelia and resins are presented in Figure 2. Most of botrallin or TMC-264 was transferred to the resins. Among them, DM-301 resin showed the most enhancing effect on botrallin and TMC-264 production. When treated with DM-301 resin, the total yields of botrallin and TMC-264 were 27.98 mg/L and 33.86 mg/L, which were 1.43-fold and 8.98-fold higher in comparison with the control (19.52 mg/L and 3.77 mg/L), respectively. According to the “like dissolves like” principle, the resins DM-301 and ADS-17 have similar polarity to botrallin and TMC-264, thus make them suitable for adsorbing small and middle-polarity molecules [29]. S-8 resin maybe adsorbed the biosynthetic precursors of botrallin to lead to decrease botrallin production that needs to be further studied. DM-301 resin was selected for the following studies on its addition concentration, addition time and incubation period for the production of botrallin and TMC-264 in liquid culture of *Hyalodendriella* sp. Ponipodef12.

### 2.3. Effects of DM-301 Resin on Mycelial Growth and Production of Botrallin and TMC-264

**Figure 3:**
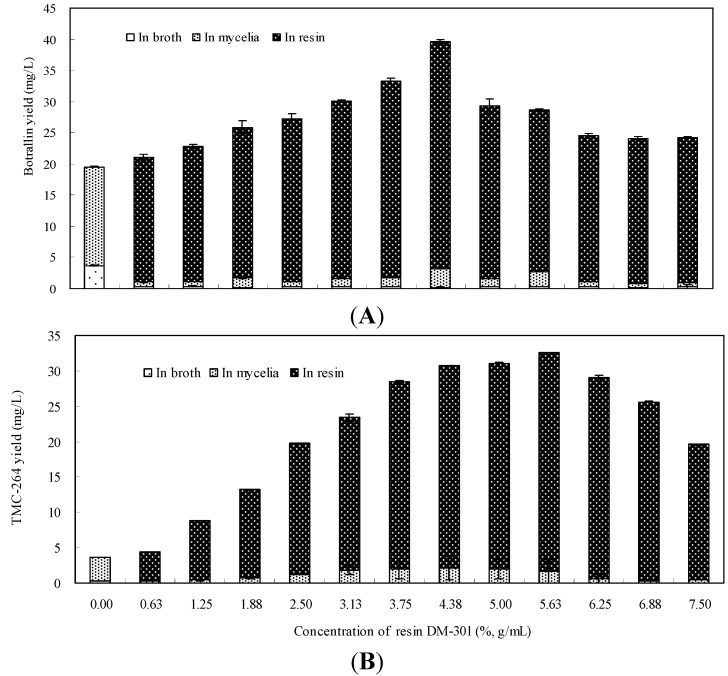
Effects of DM-301 resin at its different concentrations on production of botrallin (**A**) and TMC-264 (**B**) along with their distribution in the broth, mycelia and resins in liquid culture of *Hyalodendriella* sp. Ponipodef12 (error bars for standard deviations, *n* = 3).

DM-301 resin was added to the medium from 0% (control) to 7.50% (g/mL) in order to determine the most suitable concentration for the mycelial growth, production of botrallin and TMC-264 in liquid culture of *Hyalodendriella* sp. Ponipodef12 (Table 2). When the concentration of DM-301 resin addition was increased, the amount of adsorbed botrallin and TMC-264 gradually increased too. Botrallin production reached the highest level (39.58 mg/L) which was 2.02-fold higher than the control (19.55 mg/L) when DM-301 resin was added in the medium at 4.38% loading. Similarly, TMC-264 production reached the highest value (34.13 mg/L) which was 9.28-fold higher than the control (3.68 mg/L) when DM-301 resin was added in the medium at 5.63% loading. The percentages of both botrallin and TMC-264 adsorbed in DM-301 resin were between 88% and 99%, only 0 to 2% was found in the medium, and 1 to 12% remained in the mycelia (Figure 3).

When DM-301 resin was added in the medium at 4.38%, the total yield of botrallin plus TMC-264 reached the highest level (72.10 mg/L) which was 3.10-fold higher than the control (23.23 mg/L), so we conclude that 4.38% of DM-301 resin in the medium is sufficient to achieve the maximal recovery of botrallin and TMC-264 from the liquid culture, and was also optimal for improving the production of botrallin and TMC-264.

**Table 2:** Effects of the DM-301 resin on mycelial growth, production of botrallin and TMC-264 in liquid culture of *Hyalodendriella* sp. Ponipodef12.

Conc. of DM-301 Resin (%, g/mL)	Mycelia Biomass (g dw/L)	Botrallin Yield (mg/L)	TMC-264 Yield (mg/L)	Yield of Botrallin Plus TMC-264 (mg/L)
0.00	7.34 ± 0.08 a	19.55 ± 0.06 j	3.68 ± 0.05 k	23.23 ± 0.07 k
0.63	7.36 ± 0.01 a	21.02 ± 0.42 i	4.40 ± 0.12 j	25.42 ± 0.32 j
1.25	7.34 ± 0.03 a	22.80 ± 0.38 h	8.87 ± 0.07 i	31.67 ± 0.44 i
1.88	7.35 ± 0.01 a	25.83 ± 1.03 f	13.16 ± 0.17 h	39.00 ± 0.88 h
2.50	7.35 ± 0.04 a	27.30 ± 0.79 e	19.72 ± 0.03 g	47.02 ± 0.78 f
3.13	7.36 ± 0.01 a	30.12 ± 0.12 c	24.08 ± 0.57 f	54.20 ± 0.65 d
3.75	7.38 ± 0.03 a	33.27 ± 0.46 b	30.13 ± 0.25 c	63.40 ± 0.21 b
4.38	7.36 ± 0.03 a	39.58 ± 0.34 a	32.52 ± 0.28 b	72.10 ± 0.45 a
5.00	7.18 ± 0.17 b	29.33 ± 1.06 cd	32.76 ± 0.17 b	62.09 ± 1.17 c
5.63	7.17 ± 0.07 b	28.64 ± 0.14 d	34.13 ± 0.22 a	62.77 ± 0.04 bc
6.25	7.13 ± 0.02 b	24.58 ± 0.23 g	28.78 ± 0.39 d	53.36 ± 0.50 d
6.88	7.12 ± 0.02 b	24.15 ± 0.27 g	25.63 ± 0.02 e	49.78 ± 0.26 e
7.50	7.12 ± 0.03 b	24.22 ± 0.34 g	19.61 ± 0.09 g	43.83 ± 0.26 g

*Notes*: CK, without addition of the test resin; the values are expressed as means ± standard deviations (*n* = 3). Different letters (*i.e*., a, b, c, …) indicate significant differences among the treatments of DM-301 resin at its concentrations in each column at *p* = 0.05 level.

There was no significant effect on mycelial growth with DM-301 resin added to the medium at concentrations ranging from 0% to 4.38% (g/mL), the mycelia biomass was relatively low when DM-301 resin was added at concentrations from 5.00% to 7.50%.

In this study, the yields of botrallin and TMC-264 reached their maximum values and then declined with increasing DM-301 resin concentration. Our results are consistent with previous reports on the production of a prodigiosin-like red pigment [30], dynemcin A [31], teicoplanin [32], and cercosporamide [33]. Interestingly, when the resin concentrations were increased, no declines in product concentrations were observed either in the previous reports on the production of rubradirin [34], beauvericin [26], and diepoxin ζ [27].

### 2.4. Effects of DM-301 Resin Addition and Incubation Time on Mycelial Growth and Production of Botrallin and TMC-264

The effects of DM-301 resin addition time and incubation period on mycelial growth and production of botrallin and TMC-264 are shown in Table 3.

**Table 3:** Effects of addition and incubation time of DM-301 resin on mycelial growth and production of botrallin and TMC-264 of *Hyalodendriella* sp. Ponipodef12 in liquid culture.

Resin Addition Time (day)	Incubation Time/Harvest Time (day/day)	Mycelia Biomass (g dw/L)	Botrallin Yield (mg/L)	TMC-264 Yield (mg/L)	Yield of Botrallin Plus TMC-264 (mg/L)
CK	0/20	6.20 ± 0.05 e	7.26 ± 0.08 l	3.88 ± 0.05 k	11.14 ± 0.04 m
	0/22	6.87 ± 0.04 c	17.17 ± 0.06 k	4.85 ± 0.02 j	22.02 ± 0.07 k
	0/24	7.24 ± 0.01 a	21.19 ± 0.02 j	5.08 ± 0.07 j	26.26 ± 0.05 i
	0/26	7.35 ± 0.01 a	22.97 ± 0.38 j	5.61 ± 0.06 j	28.58 ± 0.43 h
	0/28	7.39 ± 0.01 a	22.49 ± 0.24 i	5.00 ± 0.18 j	27.48 ± 0.14 hi
	0/30	7.34 ± 0.01 a	20.21 ± 0.03 j	4.06 ± 0.07 k	24.27 ± 0.07 j
	0/32	6.93 ± 0.07 bc	19.37 ± 0.17 j	3.39 ± 0.03 kl	22.76 ± 0.15 k
	0/34	6.87 ± 0.19 c	17.43 ± 0.82 k	3.14 ± 0.23 l	20.57 ± 0.78 l
	0/36	5.69 ± 0.13 e	17.06 ± 0.29 k	2.81 ± 0.07 m	19.87 ± 0.36 l
20	2/22	6.64 ± 0.11 d	33.46 ± 0.99 g	13.86 ± 0.24 i	47.32 ± 1.17 g
	4/24	7.18 ± 0.15 b	36.07 ± 0.67 f	33.44 ± 0.11 e	69.51 ± 0.78 e
	6/26	6.93 ± 0.02 bc	41.15 ± 0.36 de	37.06 ± 0.58 c	78.21 ± 0.84 c
	8/28	6.91 ± 0.05 bc	42.74±0.63 cd	34.79±0.24 de	77.53±0.86 c
	10/30	6.92 ± 0.05 bc	42.48 ± 0.36 cd	35.08 ± 0.77 d	77.56 ± 1.10 c
22	2/24	7.38 ± 0.02 a	45.83 ± 0.43 c	22.87 ± 1.10 g	68.70 ± 1.12 e
	4/26	7.35 ± 0.24 a	50.66 ± 0.36 a	37.58 ± 0.07 c	88.24 ± 0.30 b
	6/28	7.34 ± 0.02 a	49.86 ± 0.11 a	37.24 ± 0.06 c	87.11 ± 0.16 b
	8/30	7.32 ± 0.01 a	49.95 ± 0.30 a	37.43 ± 0.10 c	87.38 ± 0.36 b
	10/32	6.98 ± 0.06 bc	49.24 ± 0.38 ab	36.77 ± 0.11 cd	86.01 ± 0.42 b
24	2/26	7.34 ± 0.03 a	40.82 ± 0.26 e	27.69 ± 0.22 f	68.50 ± 0.46 e
	4/28	7.55 ± 0.06 a	51.47 ± 0.37 a	45.85 ± 0.14 b	97.32 ± 0.49 a
	6/30	7.33 ± 0.04 a	49.44 ± 0.10 ab	47.74 ± 0.46 a	97.18 ± 0.56 a
	8/32	6.88 ± 0.06 c	48.80 ± 0.47 b	47.10 ± 0.08 a	95.89 ± 0.48 a
	10/34	6.85 ± 0.07 c	48.88 ± 0.40 b	47.07 ± 0.03 a	95.95 ± 0.38 a
26	2/28	7.36 ± 0.04 a	28.03 ± 1.37 i	18.01 ± 0.12 h	46.04 ± 1.43 g
	4/30	7.15 ± 0.05 b	31.76 ± 0.83 h	27.04 ± 0.19 f	58.79 ± 0.95 f
	6/32	6.88 ± 0.02 c	37.27 ± 0.05 f	36.58 ± 0.17 cd	73.85 ± 0.12 d
	8/34	6.78 ± 0.05 cd	43.49 ± 0.15 c	35.48 ± 0.51 d	78.97 ± 0.65 c
	10/36	5.21 ± 0.03 f	43.68 ± 0.01 c	35.18 ± 0.50 d	78.85 ± 0.50 c

*Notes*: CK, without addition of the test resin; the concentration of resin DM-301 in medium was 4.38% (g/mL); the values are expressed as means ± standard deviations (*n* = 3). Different letters (*i.e*., a, b, c, …) indicate significant differences among the treatments including addition and incubation time in each column at *p* = 0.05 level.

The addition of DM-301 resin to the culture broth on day 24 and harvesting on days 26, 28, 30, 32 and 34, respectively, resulted in mycelial growth equal to that of the control, while botrallin and TMC-264 levels increased steadily as the harvest time was extended from 26 days to 28 days, and from 26 to 30, respectively, and then reached a stable level. The maximum yield of botrallin reached 51.47 mg/L, which was 2.29-fold higher than the control (22.49 mg/L), after adding 4.38% (w/v) DM-301 resin into the culture broth on day 24 of culture and allowing a 4 days period for adsorption. The maximum yield of TMC-264 reached 47.74 mg/L, which was 11.76-fold higher than the control (4.06 mg/L), after adding 4.38% (w/v) DM-301 resin into the culture broth on day 24 of culture and allowing a period of 6 days for adsorption.

Considering production of the two dibenzo-α-pyrones, the highest yield of botrallin plus TMC-264 reached 97.32 mg/L, which was 3.54-fold higher than the control (27.48 mg/L), obtained by adding DM-301 resin on day 24 of culture and allowing a period of 4 days for adsorption.

As shown in Table 3, if DM-301 resin was added early (*i.e*., the addition time occurred on day 20 or day 22), it possibly adsorbed more nutrients and thus caused a decrease of botrallin and TMC-264 biosynthesis. If DM-301 resin was added to the culture system at the later stages (*i.e*., the addition happened on day 26), the cell viability of the fungus was low, and biosynthesis of botrallin and TMC-264 decreased, though they were partly adsorbed by the resin which only acted as an storage but did not influence the synthesis of botrallin and TMC-264. The reason for this phenomenon needs to be further verified. Similar results were observed in the previous reports such as beauvericin production in mycelial culture of *Fusarium redolens* Dzf2 [26], trichodimerol production in fermentation culture of *Penicillium* *chrysogenum* [35], pristinamycin production in fermentation culture of *Streptomyces pristinaespiralis* [36], and production of anthraquinones in cell suspension culture of *Morinda elliptica* [37].

## 3. Experimental Section

### 3.1. Endophytic Fungus and Culture Conditions

The endophytic fungus *Hyalodendriella* sp. Ponipodef12 (GenBank accession number HQ731647) was isolated from the healthy stems of the “Neva” hybrid of *Populus deltoides* Marsh × *P. nigra* L in our previous study [38]. It was stored both on PDA slants at 4 °C and in 40% glycerol at −70 °C in the Herbarium of the College of Agronomy and Biotechnology, China Agricultural University (Beijing, China). The fungus was cultured on PDA (potato 200 g/L, dextrose 20 g/L and agar 20 g/L) medium in Petri dishes at 25 °C for 10 days. For seed culture, four plugs of agar medium (0.4 × 0.4 cm) with fungal cultures were inoculated in each 250-mL Erlenmeyer flask containing 100 mL potato dextrose broth (PDB) medium, and incubated on a rotary shaker at 150 rpm and 25 °C for 5 days. For fermentation culture, about 14 mycelia pellets were inoculated in each 250 mL Erlenmeyer flask containing 80 mL PDB medium, and incubated on a rotary shaker at 150 rpm and 25 °C.

### 3.2. Macroporous Resin Selection and Preparation

Thirteen nonionic polystyrene resins, which were purchased from Tianjin Haiguang Chemical Company of China (Tianjin, China), were initially examined as the adsorbents for *in situ* adsorption of botrallin and TMC-264 in mycelial liquid culture of *Hyalodendriella* sp. Ponipodef12. Their chemical and physical properties, including polymer chemistry, polarity, particle size, surface area, pore size and moisture content are listed in Table 4. Prior to use, the resins were soaked in methanol for 24 h at room temperature and then washed thoroughly with distilled water in a Buchner funnel and dried in an oven at 35–40 °C to constant dry weight (dw). The dried resin was wrapped with 30 μm-pore nylon cloth (Yanpai Chemical Company, Shanghai, China) into small bags of 0.5–6.0 g. The resin bags were suspended in distilled water and autoclaved at 121 °C for 20 min before being added to the culture broth at the designated time.

**Table 4:** Chemical and physical properties of the macroporous resins employed.

Macroporous Resin	Polymer Chemistry	Polarity	Particle Size (mm)	Surface Area (m^2^/g)	Average pore Diameter (nm)	Moisture Content (%)
X-5	Polystyrene	Non-polar	0.30–1.25	500–600	29–30	50.13 ± 0.17
D-101	Polystyrene	Non-polar	0.30–1.25	480–530	9–11	67.89 ± 0.27
HPD-100	Polystyrene	Non-polar	0.30–1.25	≥650	8–9	68.39 ± 0.48
D1300	Polystyrene	Non-polar	0.30–1.25	≥600	9–10	68.27 ± 0.57
D3520	Polystyrene	Non-polar	0.30–1.25	480–520	8.5–9	71.56 ± 0.18
AB-8	Polystyrene	Weak-polar	0.30–1.25	480–520	13–4	68.33 ± 0.61
DM130	Polystyrene	Weak-polar	0.30–1.25	500–550	9–10	66.00 ± 0.32
DS-8	Polystyrene	Weak-polar	0.30–1.25	500–550	12–14	63.55 ± 0.10
DM-301	Polystyrene	Middle-polar	0.30–1.25	≥330	14–17	65.48 ± 1.32
ADS-17	Polystyrene	Middle-polar	0.30–1.25	90–150	25–30	55.16 ± 1.09
S-8	Polystyrene	Polar	0.30–1.25	100–200	28–30	67.12 ± 0.35
NKA-9	Polystyrene	Polar	0.30–1.25	250–290	15.5–16.5	70.22 ± 0.23
DA-201	Polystyrene	Polar	0.30–1.25	≥200	10–13	70.64 ± 0.52

Note: The information was provided by the manufacturers, except for the moisture content.

### 3.3. In Situ Adsorption Culture

For screening the optimal resin from among the 13 candidates, 4.0 g of each resin in a nylon bag was added to the mycelial culture flask (80 mL medium in 250 mL flask) on day 22 of culture, so the corresponding concentration of resin in the medium was 5.00% (g/mL). The cultures were harvested on day 30. For determining the suitable concentration of the selected resin DM-301, different quantities (0, 0.5, 1.0, 1.5, 2.0, 2.5, 3.0, 3.5, 4.0, 4.5, 5.0, 5.5, 6.0 g) of DM-301 resin were added in 80 mL medium (their corresponding concentations were 0%–7.50%, g/mL) on day 22 of culture. The cultures were harvested on day 30. For obtaining the optimal addition and incubation time of DM-301 resin, 4.38% (3.5 g in 80 mL medium) of resin was added into the medium on days 20, 22, 24, and 26 of culture, respectively. The cultures were harvested every two days after each addition time. All treatments were performed in triplicate.

### 3.4. Analytical Procedures

The mycelia of *Hyalodendriella* sp. Ponipodef12 were separated from the liquid medium by filtration under vacuum and rinsed thoroughly with distilled water, and then dried at 60 °C in an oven to constant dry weight (dw). Botrallin and TMC-264 were extracted from either the dry mycelial powder (10 mg/mL) or the resin (100 mg/mL) with ethyl acetate in an ultrasonic bath (three times, 60 min each). After removal of the solid by filtration, the liquid extract was evaporated to dryness and redissolved in 1 mL of methanol. For analysis of botrallin and TMC-264 yield in medium, 5 mL of the culture broth was evaporated to dryness and extracted with 5 mL of ethyl acetate, and the liquid extraction was then evaporated to dryness and redissolved in 1 mL of methanol.

**Figure 4:**
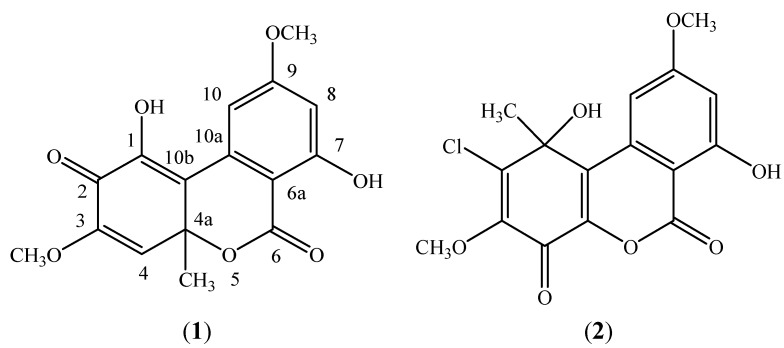
The structures of botrallin (**1**) and TMC-264 (**2**).

The botrallin and TMC-264 standards were isolated and identified from the fermentation cultures of the endophytic fungus *Hyalodendriella* sp. Ponipodef12 in our previous studies [12,14]. Their structures are shown in Figure 4. The content of botrallin and TMC-264 was analyzed on a high performance liquid chromatography (HPLC) system (Shimadzu, Kyoto, Japan), which consisted of two LC-20AT solvent delivery units, an SIL-20A autosampler, a SPD-M20A photodiode array detector, and CBM-20Alite system controller. A reversed-phase Ultimate TM XB C_18_ column (250 mm × 4.6 mm, 5 μm, Welch Materials, Inc., Ellicott, MD, USA) was used for separation by using a mobile phase of methanol-H_2_O (60:40, v/v) at a flow rate of 1 mL/min. The temperature was maintained at 40 °C, and UV detection at 234 nm. The sample injection volume was 10 μL. The LC-solution multi-PDA workstation was employed to acquire and process chromatographic data. The two compounds were detected and quantified with the standards obtained in the study.

### 3.5. Statistical Analysis

All tests were carried out in triplicate, and the results were represented by their mean values and the standard deviations (SD). The data were submitted to analysis of variance (one-way ANOVA) to detect significant differences by PROC ANOVA of SAS version 8.2. The term significant has been used to denote the differences for which *p* ≤ 0.05.

## 4. Conclusions

In this study, thirteen macroporous adsorption resins with different polarities were evaluated for their enhancing effects on botrallin and TMC-264 production in mycelial liquid culture of *Hyalodendriella* sp. Ponipodef12. DM-301 resin was shown to most effectively enhance production of botrallin and TMC-264 among the tested resins. The highest botrallin yield reached 51.47 mg/L which was 2.29-fold higher than the control (22.49 mg/L), and was achieved by adding DM-301 resin at 4.38% (g/mL) to the culture broth on day 24 of culture and allowing a period of 4 days for adsorption. The highest yield of TMC-264 reached 47.74 mg/L, which was 11.76-fold higher than the control (4.06 mg/L), after adding DM-301 resin at 4.38% (w/v) to the culture broth on day 24 of culture and allowing 6 days for adsorption.

For the thirteen representative macroporous resins, we should test each resin at different concentrations during fermentation to obtain an appropriate combination of the resin and its concentration along with the addition and incubation time needed to enhance production of botrallin and TMC-264, but this would be time-consuming and lead to a large amount of labor. In addition, as the number of the adsorption resins employed in this study was very limited, there might be other more appropriate resins that were not screened. Nevertheless, we can state that *in situ* resin adsorption is an effective strategy for enhancing the production of botrallin and TMC-264, and also facilitating their recovery from mycelial liquid culture of *Hyalodendriella* sp. Ponipodef12. This is the first report on the use of *an in situ* product removal strategy for improving botrallin and TMC-264 production in mycelial liquid culture of this endophytic fungus. The enhancing effects may be the result of elimination of feedback inhibition, overcoming product degradation, and sequestering products to avoid their toxicity to the producing organism [23,24,36]. The results demonstrated the potential application of *in situ* solid-phase adsorption strategy for producing botrallin and TMC-264 in mycelial liquid culture of *Hyalodendriella* sp. Ponipodef12. In this study, we just considered four variables by employing macroporous resin adsorption during fermentation such as the type, concentration, addition time and incubation period of resin. Other variables such as temperature, pH value, fermentation medium, combination effects of two or more resins, adsorption and desorption kinetics, an bioreactor system for large-scale production by utilizing resin adsorption of botrallin and TMC-264 need to be studied in detail [24].
